# Functional expression of the proton sensors ASIC1a, TMEM206, and OGR1 together with BK_Ca_ channels is associated with cell volume changes and cell death under strongly acidic conditions in DAOY medulloblastoma cells

**DOI:** 10.1007/s00424-024-02964-7

**Published:** 2024-04-16

**Authors:** Karolos-Philippos Pissas, Stefan Gründer, Yuemin Tian

**Affiliations:** Institute of Physiology, RWTH Aachen University, Pauwelsstraße 30, 52074 Aachen, Germany

**Keywords:** Cell death, Extracellular acidity, Cell volume regulation, Tumour microenvironment, Medulloblastoma, Ion channels

## Abstract

Fast growing solid tumors are frequently surrounded by an acidic microenvironment. Tumor cells employ a variety of mechanisms to survive and proliferate under these harsh conditions. In that regard, acid-sensitive membrane receptors constitute a particularly interesting target, since they can affect cellular functions through ion flow and second messenger cascades. Our knowledge of these processes remains sparse, however, especially regarding medulloblastoma, the most common pediatric CNS malignancy. In this study, using RT-qPCR, whole-cell patch clamp, and Ca^2+^-imaging, we uncovered several ion channels and a G protein-coupled receptor, which were regulated directly or indirectly by low extracellular pH in DAOY and UW228 medulloblastoma cells. Acidification directly activated acid-sensing ion channel 1a (ASIC1a), the proton-activated Cl^−^ channel (PAC, ASOR, or TMEM206), and the proton-activated G protein-coupled receptor OGR1. The resulting Ca^2+^ signal secondarily activated the large conductance calcium-activated potassium channel (BK_Ca_). Our analyses uncover a complex relationship of these transmembrane proteins in DAOY cells that resulted in cell volume changes and induced cell death under strongly acidic conditions. Collectively, our results suggest that these ion channels in concert with OGR1 may shape the growth and evolution of medulloblastoma cells in their acidic microenvironment.

## Introduction

Medulloblastoma (MB) is the most common solid tumor in pediatric patients [14]. It constitutes a fast-growing infratentorial embryonic tumor with a high metastatic potential. Even though overall survival of patients with MB has drastically improved over the last decades, many children still suffer from long-term side effects of the treatment or of the tumor itself [26, 43]. Therefore, further research is necessary to better understand MB pathophysiology and discover novel treatment targets. The acidic and hypoxic tumor microenvironment (TME) of MBs potentially constitutes such a target, as the tumor cells must adapt to these conditions to avoid apoptosis and to promote proliferation. A variety of plasma membrane proteins, such as ion channels and transporters, are involved in this adaptation [3, 4].

Membrane-resident proton sensors are a first line of detection of the acidic TME. Recently, we identified and characterized the acid-sensing ion channel 1a (ASIC1a) in DAOY MB cells [42] and found that the activation of ASIC1a by extracellular acidification may induce an uncanonical pathway of necroptosis [10, 50, 52] in MB cells [42].

The functions of the proton-activated Cl^−^ channel TMEM206, which has only recently been molecularly characterized [48, 58], are only beginning to emerge. It has previously been linked to cell volume changes under acidic pH [48, 51]. Since it requires lower pH stimulation than ASICs, emerging evidence points towards additional functions in strongly acidic intracellular compartments [40]. A recent study unveiled the key role of TMEM206 in macropinosome resolution [59]. Macropinocytosis is of central importance for cancer cells, as they employ it to take up nutrients and proliferate in hypoxic, acidic and nutrient poor TMEs [46].

The ovarian cancer G protein-coupled receptor (OGR1) is a proton-activated GPCR [22], which is also expressed in DAOY cells [21]. OGR1 is fully inactive at a pH of 7.8 and fully active at a pH of 6.8 [33]. It promotes proliferation and migration in many tumors, such as pancreatic cancer, prostate cancer, and colon cancer [55]. In DAOY cells, OGR1 activation increases intracellular Ca^2+^ concentration [Ca^2+^]_i_ and activates the MEK/ERK pathway under mild acidic conditions [21].

BK_Ca_ channels have been studied quite extensively in gliomas and other tumors where they are often associated with tumor proliferation and migration [1, 17, 28, 62]. BK_Ca_ and other K^+^ channels have been associated with cell volume regulation, especially regarding cell cycle progression [34, 49, 57]. But so far, BK_Ca_ channels have not been characterized in MB.

Medulloblastomas are divided into four molecular subtypes, namely WNT, SHH, group 3, and group 4 tumors. They possess distinct genetic profiles and vastly different survival rates [38, 47]. WNT tumors make up 10–15% of all MBs and have the most favorable prognosis with a 5-year survival rate of > 90%. Group 3 tumors have the worst outcome (5-year survival < 60%) and account for about 20% of MBs. SHH and group 4 tumors account for 30% and 40% of MBs, respectively, and possess intermediate survival rates. DAOY cells are a model for SHH MB, and UW228 cells are a model for WNT MB [18].

In the present study, we characterized different ionic currents elicited by acidic pH in DAOY and UW228 MB cells. Functional experiments uncovered a complex interplay between ASIC1a, TMEM206, OGR1, and BK_Ca_ in DAOY cells under acidic conditions, which induced changes in cell volume and cell death.

## Results

### Strong acidification activates TMEM206 in DAOY and UW228 cells

**Fig. 1:**
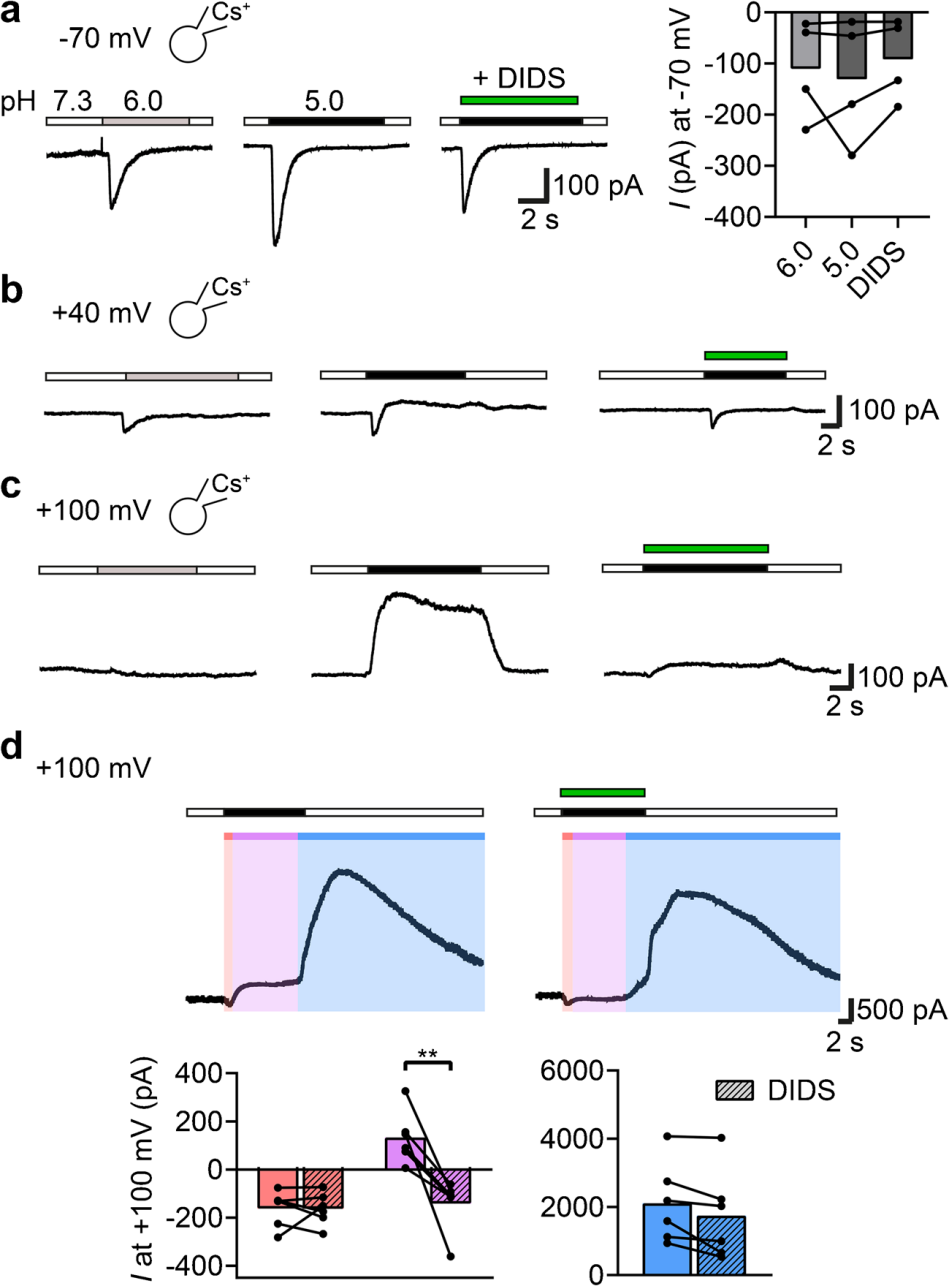
Strong acidification activates an outwardly-rectifying, DIDS-sensitive channel in DAOY cells. (**a**) Left, representative traces of a DAOY cell stimulated with pH 6.0, pH 5.0, or pH 5.0 plus 100 μM DIDS. Holding potential was -70 mV and the pipette contained Cs^+^. Right, mean amplitudes of the inward currents (*n* = 4). (**b**) As in a but at + 40 mV. (**c**) As in a but at + 100 mV. (**d**) Top, representative traces of a DAOY cell stimulated with pH 5.0 or with pH 5.0 plus DIDS. Holding potential was + 100 mV and the pipette contained no Cs^+^. Bottom, mean amplitudes of three different current types (*n* = 6), highlighted by red, pink, and blue bars, respectively. Note that the blue outward current appeared with some delay and was potentiated upon return to pH 7.3. **, *P* < 0.01 (paired Student’s t-test)

We have previously reported the expression of functional ASIC1a in DAOY cells [42]. To test for the presence of other acid-sensitive ion channels, we clamped the cells at different holding potentials (-70 mV, + 40 mV and + 100 mV) and applied pH 6.0 or pH 5.0 (Fig. 1a-c). To prevent the contamination by K^+^ channels, we initially used Cs^+^ in the pipette. At -70 mV, acidic pH elicited only a transient inward current which is carried by homomeric ASIC1a [42] and which was not sensitive to DIDS (Fig. 1a), an inhibitor of many anion channels and transporters, including TMEM206 [48, 51]. At + 40 mV, pH 5.0 but not pH 6.0 elicited an outward, DIDS-sensitive current (Fig. 1b). This current was even more prominent at + 100 mV (Fig. 1c). Without Cs^+^ in the pipette and at + 100 mV, with some delay another large outward current appeared that was potentiated upon return to neutral pH 7.3 (blue bar in Fig. 1d), most likely a K^+^ current.

**Fig. 2:**
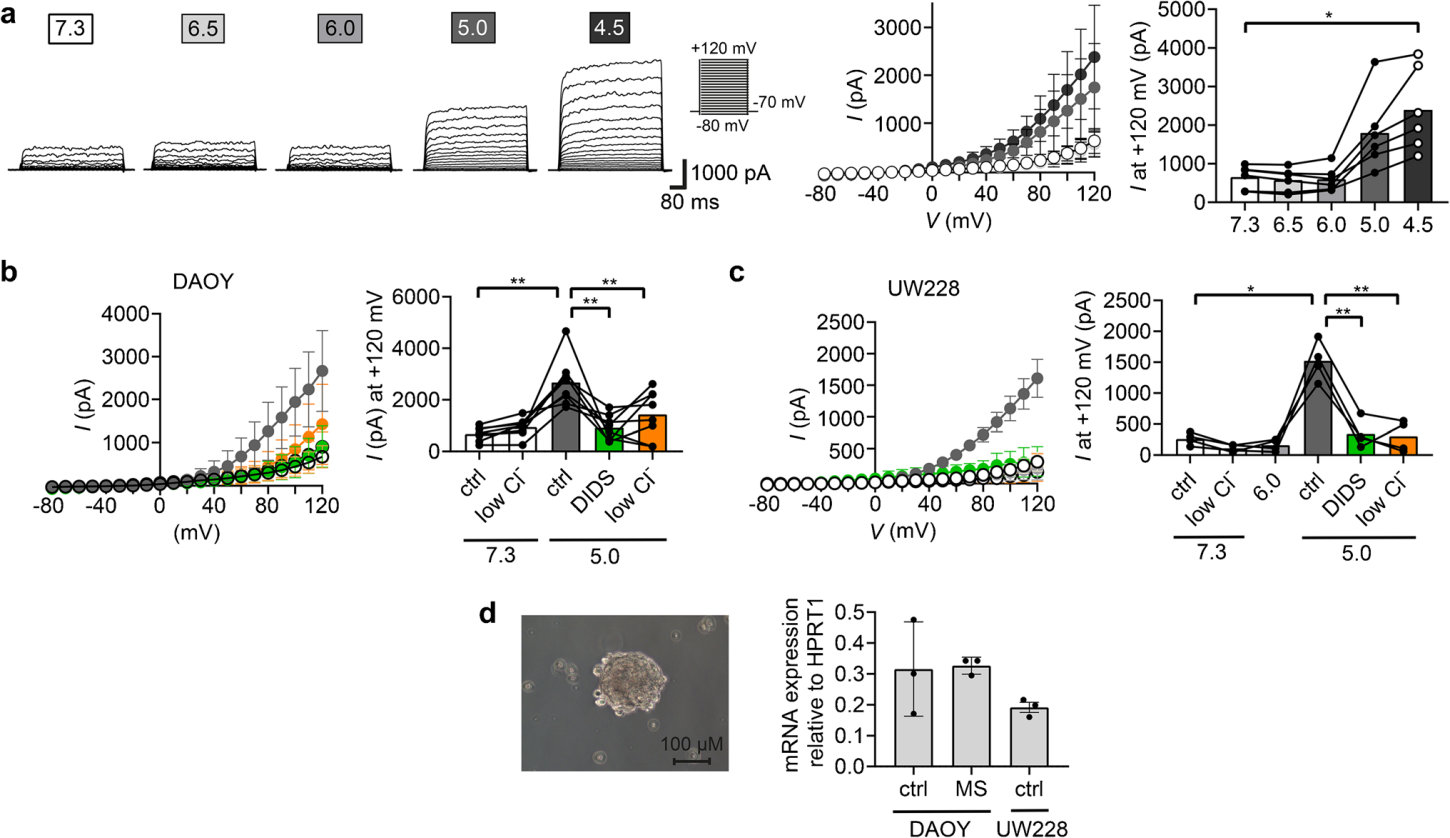
DAOY and UW228 cells express functional TMEM206 (**a**) Left, representative traces of a DAOY cell treated with different acidic pH. Middle, current–voltage (IV) curves (mean ± SD). Right, mean current amplitudes at + 120 mV (*n* = 6). (**b**) Left, IV curves (mean ± SD) of DAOY cells treated with the following 5 conditions: (i) ctrl at pH 7.3; (ii) low Cl^−^ at pH 7.3; (iii) ctrl at pH 5.0; (iv) low Cl^−^ at pH 5.0; (v) pH 5.0 plus 100 μM DIDS. Right, mean current amplitudes at + 120 mV (*n* = 8). (**c**) Left, IV-curves of UW228 cells treated with the same conditions as in b and with pH 6.0 (mean ± SD). Right, mean current amplitudes at + 120 mV (*n* = 4). (**d**) Left, light microscopy image of DAOY medullosphere cultured for 7 days. Right, mRNA expression of TMEM206 in DAOY and UW228 cells cultured as monolayers (ctrl) and in DAOY cells cultured as medullospheres (MS) for 7 days (mean ± SD). qPCR data was normalized to *HPRT1* expression levels (*n* = 3). *, *P* < 0.05; **, *P* < 0.01 (repeated measures one-way ANOVA)

We asked whether the DIDS-sensitive outward current elicited by pH 5.0 was carried by TMEM206 and stimulated DAOY cells with different acidic pH and applied a voltage step protocol (Fig. 2a). The protocol was applied 2 s after the onset of acidic stimulation, before the activation of the delayed K^+^ current recorded in Fig. 1d. Strong acidification (pH < 6.0) elicited an outwardly-rectifying current with activation kinetics, which closely resembled TMEM206 currents [48, 58]. Moreover, this current was reduced by DIDS and by an extracellular solution containing a low Cl^−^ concentration (5 mM; low Cl^−^) in both DAOY (Fig. 2b) and UW228 cells (Fig. 2c). RT-qPCR confirmed moderate expression levels of TMEM206 in both DAOY and UW228 cells (Fig. 2d). Collectively these results indicate that DAOY and UW228 cell express functional TMEM206, which is widely expressed in many cell lines with similar current amplitudes [9, 30, 44, 48, 51, 58].

Furthermore, to increase their stemness, we cultured DAOY cells for 7 days as medullospheres (MS) in suspension. The expression of TMEM206 was not altered in a DAOY MS culture.

### DAOY and UW228 cells express functional BK_Ca_ channels

**Fig. 3:**
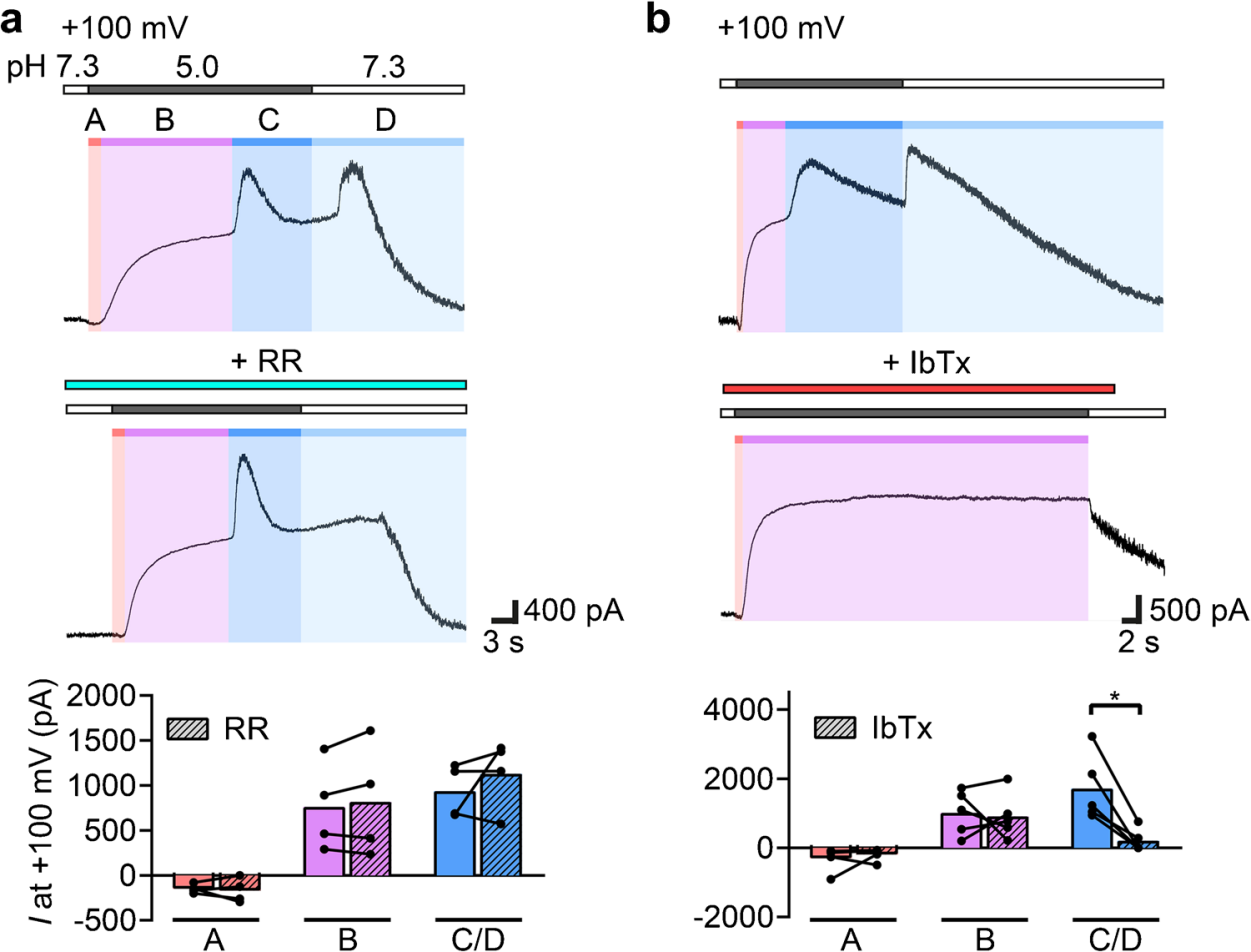
Acidic stimulation elicits four distinct currents in DAOY cells. (**a**) Top, representative traces of DAOY cells stimulated with pH 5.0 in the presence or absence of 20 μM ruthenium red (RR) at + 100 mV. Bottom, mean current amplitudes (*n* = 4). Current amplitudes were calculated by subtracting the preceding current from the maximum current in question (for example, the A current was subtracted from the maximal B current). The four distinct currents were labeled A, B, C, and D. (**b**) Same as in a, but in the presence or absence of 100 nM IbTx (*n* = 5). *, *P* < 0.05; **, *P* < 0.01 (paired Student’s t-tests)

To uncover the nature of the large delayed outward current (Fig. 1d), we more systematically analyzed currents in DAOY cells that were elicited by pH 5.0 at a strongly positive membrane potential of + 100 mV. pH 5.0 elicited a complex current pattern with one inward and two outward currents, and an additional outward current upon return to neutral pH 7.3. We labeled these four currents as A-, B-, C- and D-type, respectively (Fig. 3a). A is an inward current masked almost completely by the outward B current. Because the membrane potential in these measurements (+ 100 mV) was positive to the Na^+^ equilibrium potential (~ + 60 mV), the inward A current cannot be an ASIC current and was indeed absent when Cs^+^ was in the pipette (Fig. 1). We speculate that it arises from acid-inhibition of a K^+^ conductance. The B current had the characteristics of TMEM206 (Fig. 2). C, a strong outward current, was typically elicited with a delay of 12 ± 6 s after the start of acidic stimulation, and the outward D current was elicited quickly by switching back to pH 7.3. Unlike A and B currents, which appeared consistently in all cells, C and D currents were more variable in nature. Some cells responded with both C and D currents, while others lacked the C or the D current, respectively.

Since the acid-sensitive K2P channels TASK1/3, which are inhibited by acidic pH, have previously been characterized in DAOY cells [12], we repeated the same protocol in the presence of ruthenium red (RR), an inhibitor of TASK1/3 (Fig. 3a). All four types of currents were not affected by RR, excluding the contribution of TASK channels to these currents. Because it has previously been shown that [Ca^2+^]_i_ of DAOY cells rises in response to acidic activation of OGR1 [21, 53], we tested for the subsequent activation of the Ca^2+^-activated K^+^ channel BK_Ca_ by applying iberiotoxin (IbTx), a highly specific BK_Ca_ inhibitor. IbTx indeed fully suppressed C and D currents (Fig. 3b). Given that C and D currents were recorded variably and that they were both blocked by IbTx, we will henceforth summarize them as C/D currents. It was previously found that BK_Ca_ channels are reversibly inhibited by strong extracellular acidification [61]. Therefore, we hypothesize that BK_Ca_ was partially inhibited at pH 5.0 and that the D current corresponds to the disinhibition of BK_Ca_ channels upon return to neutral extracellular pH.

**Fig. 4:**
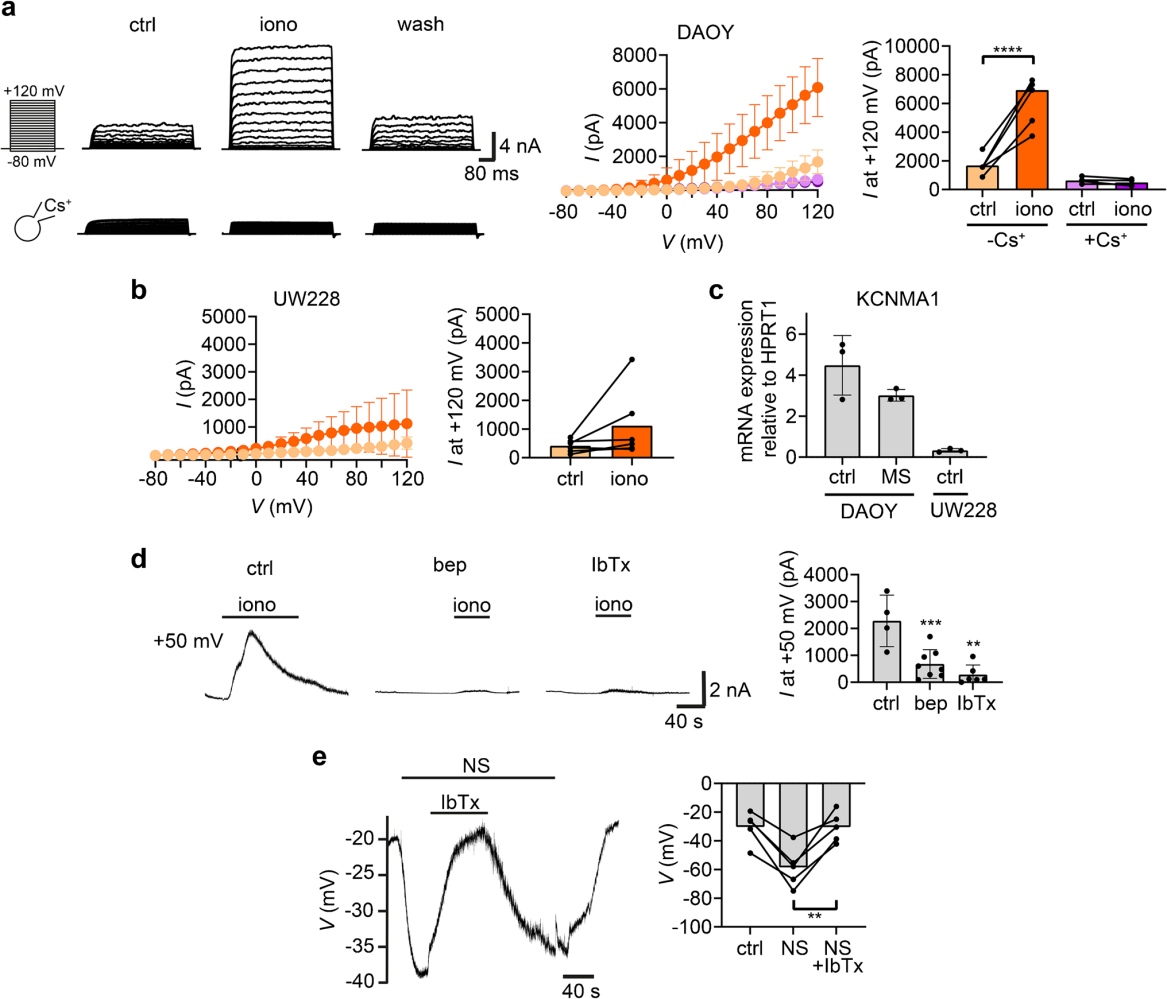
DAOY cells and UW228 cells express functional BK_Ca_ channels.** (a)** Left, representative traces of a DAOY cell before (ctrl), during (iono) and after (wash) treatment with 1 μM ionomycin. Recordings were made with a control (ctrl) or pipette solution containing Cs^+^. Middle, IV curves (mean ± SD). Right, mean current amplitudes at + 120 mV (n ≥ 4). **(b)** As in a, but with UW228 cells (*n* = 6). **(c)** mRNA expression of *KCNMA1* in DAOY and UW228 cells cultured with a pH 7.4 medium (ctrl) and in DAOY cells cultured as MS for 7 days. qPCR data was normalized to *HPRT1* expression levels (mean ± SD; *n* = 3). **(d)** Left, representative traces of DAOY cells treated with the following 3 conditions at + 50 mV: 1 μM ionomycin, 1 μM ionomycin plus 20 μM bepridil (bep), 1 μM ionomycin plus 100 nM iberiotoxin (IbTx). Right, summary of current amplitudes (mean ± SD; n ≥ 4). **(e)** Left, representative trace of a DAOY cell treated with 10 μM NS-11021 (NS) and 100 nM IbTx. Right, mean membrane potentials (*n* = 5). **, *P* < 0.01; ***, *P* < 0.001; ****, *P* < 0.0001 (paired Student’s t-tests)

To further characterize C/D currents, we used the ionophore ionomycin (iono) to unselectively activate Ca^2+^-activated channels, and a voltage step protocol, depolarizing DAOY cells from a holding potential of -70 mV to voltages ranging from -80 mV to + 120 mV in 10 mV steps of 500 ms duration each (Fig. 4a). In the presence of ionomycin, there was indeed a prominent outwardly rectifying current, which was blocked by Cs^+^ in the pipette and which had a reversal potential of ~ -49 mV, consistent with a prominent contribution by a Ca^2+^-activated K^+^ channel. Using the same experimental protocol for UW228 cells, we detected outwardly rectifying currents with an amplitude > fivefold smaller at + 120 mV (Fig. 4b). RT-qPCR confirmed that both DAOY and UW228 cells express BK channels (*KCNMA1*), although expression in UW288 cells was low (Fig. 4c), consistent with the small amplitude of outward currents in these cells. BK expression was not significantly altered in a DAOY MS culture.

We corroborated that the Ca^2+^-activated K^+^ current was carried by BK_Ca_ using two inhibitors. DAOY cells were clamped at + 50 mV and ionomycin was applied to increase [Ca^2+^]_i_. Pre-incubation with bepridil (bep), an unspecific inhibitor of many K^+^ channels including BK_Ca_ [25], or with IbTx, a highly specific BK_Ca_ inhibitor (Fig. 4d) almost completely suppressed the outwardly-rectifying K^+^-current (bep, *p* = 0.0015; IbTx, *p* = 0.0003). As further proof for functional BK_Ca_ expression in DAOY cells, we used current clamp. As previously reported [42], the resting membrane potential of DAOY cells was strongly depolarized to -30.32 mV ± 11.12 mV. Application of the specific BK_Ca_-activator NS-11201 (NS) [6] consistently hyperpolarized the cells to -58.56 mV ± 14.02 mV (Fig. 4e), likely through a K^+^ efflux. This hyperpolarization was reversed by IbTx (Fig. 4e).

### Acidification elevates [Ca^2+^]_i_ in DAOY cells but not in UW228 cells

**Fig. 5:**
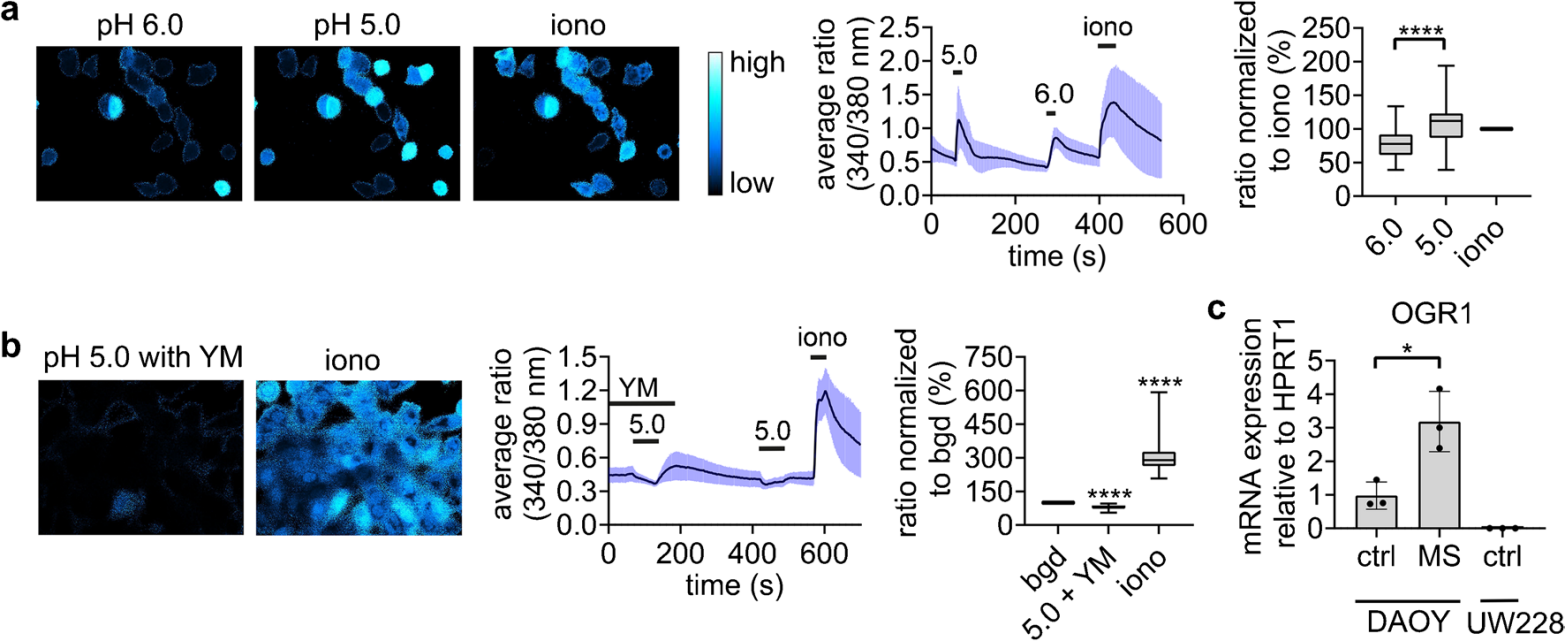
DAOY cells express functional OGR1.** (a)** Left, representative images of Ca^2+^-imaging experiments. Middle, mean ± SD of all Ca^2+^-imaging experiments of DAOY cells stimulated with pH 6.0 and pH 5.0. Right, summary data normalized to the response to ionomycin (iono) (*n* = 54). **(b)** Left, representative images of Ca^2+^-imaging experiments. Middle, mean ± SD of all Ca^2+^-imaging experiments of DAOY cells stimulated with a co-application of 1 μM YM-254890 (YM) and pH 5.0. YM was preincubated for 30 min. Right, summary data normalized to background (bgd) (*n* = 108). **(c)** mRNA expression of OGR1 in DAOY and UW228 cells cultured with a pH 7.4 medium (ctrl) and in DAOY cells cultured as MS for 7 days. qPCR data was normalized to *HPRT1* expression levels (*n* = 3). Iono was used as a positive control for all Ca^2+^-imaging experiments. Summary data are shown as box plots (a, b) or as mean ± SD (c). Statistical analysis was performed with Wilcoxon signed-ranked tests (a, b) and a repeated measures one-way ANOVA (c). *, *P* < 0.05; **, *P* < 0.01; ***, *P* < 0.001, ****, *P* < 0.0001

We next asked how BK_Ca_ channels were activated by acidic conditions in DAOY cells. We employed ratiometric Ca^2+^-imaging to confirm that a stimulation with acidic extracellular pH (pH_e_) elevates [Ca^2+^]_i_ in DAOY cells, as it had previously been described [21, 42, 53]. At pH 5.0, [Ca^2+^]_i_ increased more strongly than at pH 6.0 (*p* < 0.0001) and to a similar extent as observed with ionomycin (Fig. 5a). [Ca^2+^]_i_ began to increase ~ 4 s after acidic stimulation, reaching its peak after ~ 12–14 s, coinciding with the delayed activation of BK currents (Figs. 1 and 3). To confirm that the increase in [Ca^2+^]_i_ in DAOY cells was mediated by a GPCR, we incubated the cells with YM-254890, a general inhibitor of G proteins [41], which fully suppressed the acid-induced Ca^2+^ signal (Fig. 5b). Its inhibitory effects were difficult to wash out (Fig. 5b). RT-qPCR confirmed that DAOY cells robustly expressed OGR1 mRNA, while UW228 cells did not (Fig. 5c). This finding explains why UW228 cells do not react to acidic pH with changes in [Ca^2+^]_i_, as we have previously reported [42], but contrasts with a previous study that reported expression of OGR1 in UW228 cells [54]. Interestingly, in DAOY medullospheres (MS), the expression of OGR1 was increased threefold (*p* = 0.0179).

### Acidification induces an initial swelling and a subsequent shrinking of DAOY cells

**Fig. 6:**
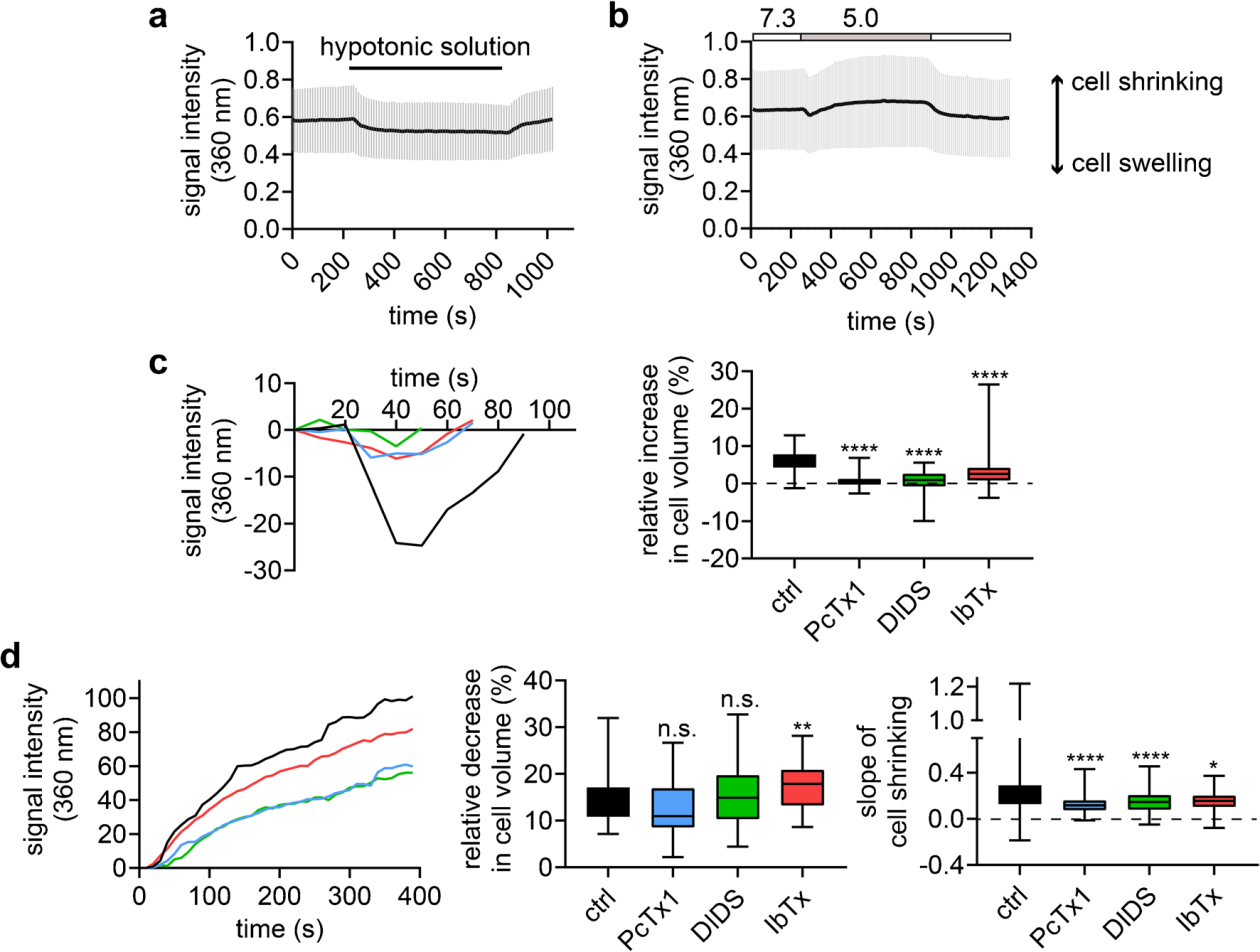
Inhibitors of ASIC1a, TMEM206 and BK_Ca_ reduce the volume changes of DAOY induced by acidic conditions.** (a)** Trace representing the change (mean ± SD) in cell volume of all DAOY cells treated with a hypotonic solution (- 50 mM). An increase in signal intensity represents relative cell shrinking, while a decrease in signal intensity represents relative cell swelling. **(b)** As in a but with a pH 5.0 stimulation. **(c)** Left, traces illustrating the mean initial, relative cell swelling of DAOY cells treated with the following 4 conditions: pH 5.0 (black trace; *n* = 94), pH 5.0 plus 100 nM PcTx1 (blue; *n* = 78), pH 5.0 plus 100 μM DIDS (green; *n* = 73), pH 5.0 plus 100 nM IbTx1 (red; *n* = 61). The beginning of the cell swelling was set to 0 s. Right, box plots summarizing the maximal relative increase in cell volume. **(d)** Left, traces illustrating the mean subsequent, relative cell shrinking of DAOY cells treated with the same conditions as in c. The beginning of the cell shrinking was set to 0 s, to better illustrate the shrinking. Middle, box plots summarizing the maximal relative decrease in cell volume relative to the initial volume. Right, box plots summarizing the slope of the cell volume decrease. *, *P* < 0.05; **, *P* < 0.01; ****, *P* < 0.0001 (Kruskal–Wallis test)

After characterizing different membrane-resident sensors of acidic pH_e_ in DAOY cells, we sought to discover whether their interplay has functional consequences. Since some of these proteins, such as TMEM206 and BK_Ca_, have been previously linked to changes in cell volume [34, 48, 51, 57], we indirectly measured changes in cell volume, using a modified Ca^2+^-imaging technique [2]. Cells loaded with the fluorescent dye Fura-2 should produce a stronger signal when they shrink and a weaker signal when they swell due to the altered local concentration of the dye. To avoid interference from Ca^2+^ signals, Fura-2 was excited at its isosbestic wavelength (358 nm). By applying a hypotonic extracellular solution which caused the cells to swell and the signal to weaken, we confirmed the validity of this method (Fig. 6a).

Application of pH 5.0 resulted in a rapid and substantial swelling of DAOY cells, followed by a slower cell shrinking, which started ~ 40 s after the onset of acidic stimulation (Fig. 6b). Inhibition of ASIC1a by psalmotoxin-1 (PcTx1) or of TMEM206 by DIDS strongly reduced the initial swelling (Fig. 6c; *P* < 0.0001), suggesting that combined activation of both channels was necessary for cell swelling. Surprisingly, inhibition of BK_Ca_ by IbTx also reduced swelling but less strongly and highly variably (Fig. 6c; *P* < 0.0001). In contrast, inhibition of ASIC1a, TMEM206, or BK_Ca_ did not reduce subsequent cell shrinking (Fig. 6d), indicating that none of the three channels was indispensable for cell volume decrease. However, the slope, reflecting the speed at which the shrinking occurred, was significantly reduced when either one of the three inhibitors was applied (*P* < 0.0001), suggesting that ASIC1a, TMEM206, and BK_Ca_ channels contributed also to the volume decrease induced by pH 5.0.

### Strong acidification induces rapid cell death in DAOY cells

**Fig. 7:**
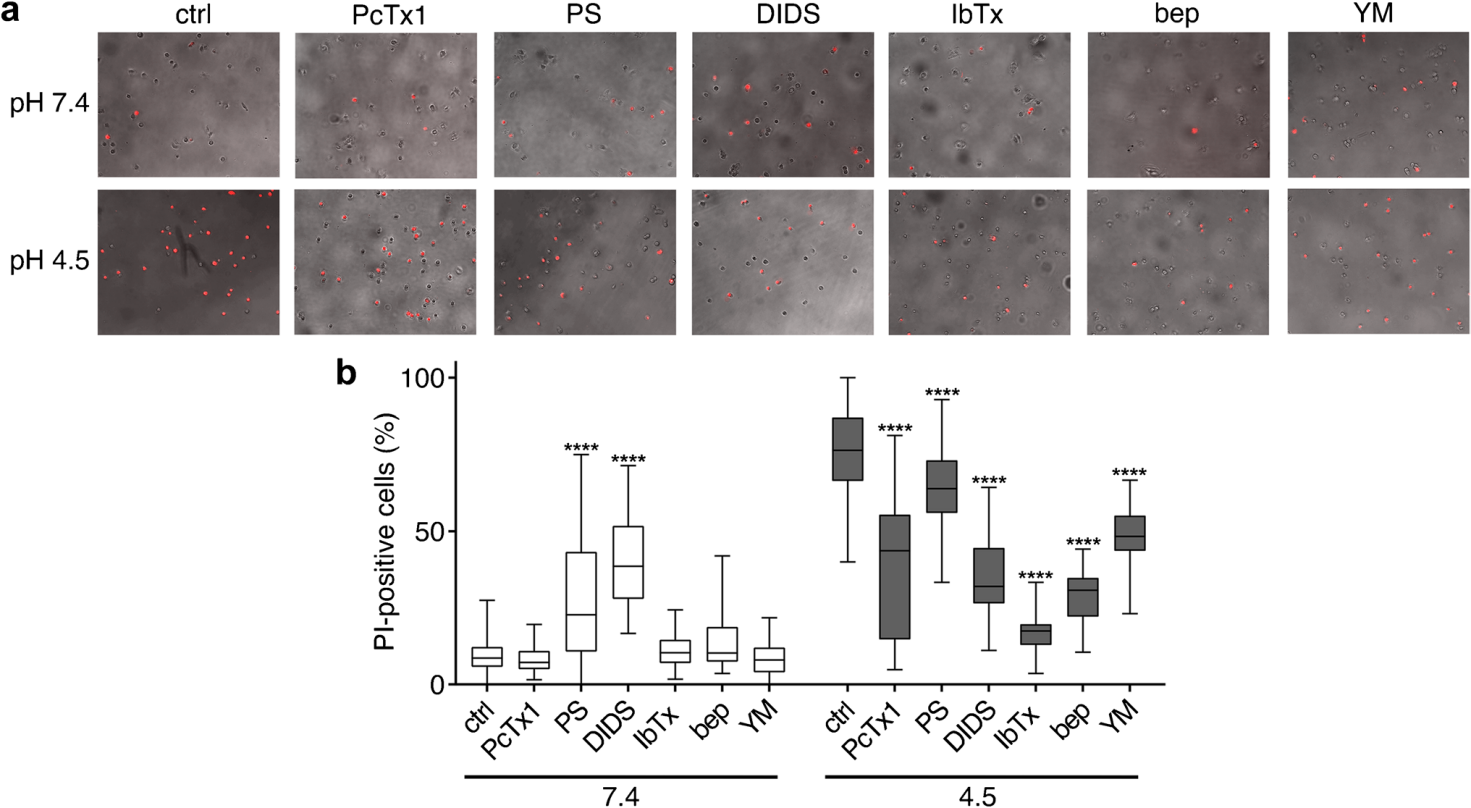
ASIC1a*,* TMEM206, BK_Ca_, and OGR1 activation induces acidotoxicity in DAOY cells.** (a)** Light microscopy images of DAOY cells treated with the following inhibitors for 2 h at pH 7.4 or at pH 4.5: 100 nM PcTx1 (*n* = 30), 100 μM pregnenolone sulfate (PS) (pH 7.4, *n* = 29; pH 4.5, *n* = 29), 100 μM DIDS (pH 7.4, *n* = 26; pH 4.5, *n* = 30), 100 nM IbTx (pH 7.4, *n* = 29; pH 4.5 = 30), 20 μM bep (pH 7.4, *n* = 29; pH 4.5, *n* = 30), and 1 μM YM (pH 7.4, *n* = 30; pH 4.5, *n* = 30). For ctrl: *n* = 107 (pH 7.4) and *n* = 110 (pH 4.5), respectively. Propidium iodide (PI) -positive cells are colored in red. (**b**) Percentage of PI-positive DAOY cells. Results are shown as box plots. ****, *P* < 0.0001 (Kruskal-Wallis test)

Finally, we employed a 2 h “shock treatment” of DAOY cells in a pH 4.5 solution, to assess the impact of strong acidification on cell viability (Fig. 7a). Propidium iodide (PI) staining of cells “shocked” with pH 4.5 revealed that DAOY cells were very vulnerable to strong acidification and that > 50% of the cells died (*P* < 0.0001 compared to pH 7.4). The addition of the following inhibitors to the “shock” solution all significantly (*P* < 0.0001) reduced cell death (Fig. 7b): PcTx1 as an ASIC1a inhibitor, pregnenolone sulfate (PS) and DIDS as TMEM206 inhibitors, IbTx and bep as BK_Ca_ inhibitors, and YM-254890 as an OGR1 inhibitor. IbTx had the strongest effect, reducing cell death almost to the levels at pH 7.4, suggesting that BK_Ca_ made a strong contribution to cell death at pH 4.5. In contrast, PcTx1 and PS and DIDS had partial effects. At pH 7.4, both PS and DIDS increased the number of PI-positive cells; the reason for this increased cell death is currently unknown.

### TMEM206, BK_Ca_*, *and OGR1 show a distinct mRNA expression pattern in MB tissue

**Fig. 8:**
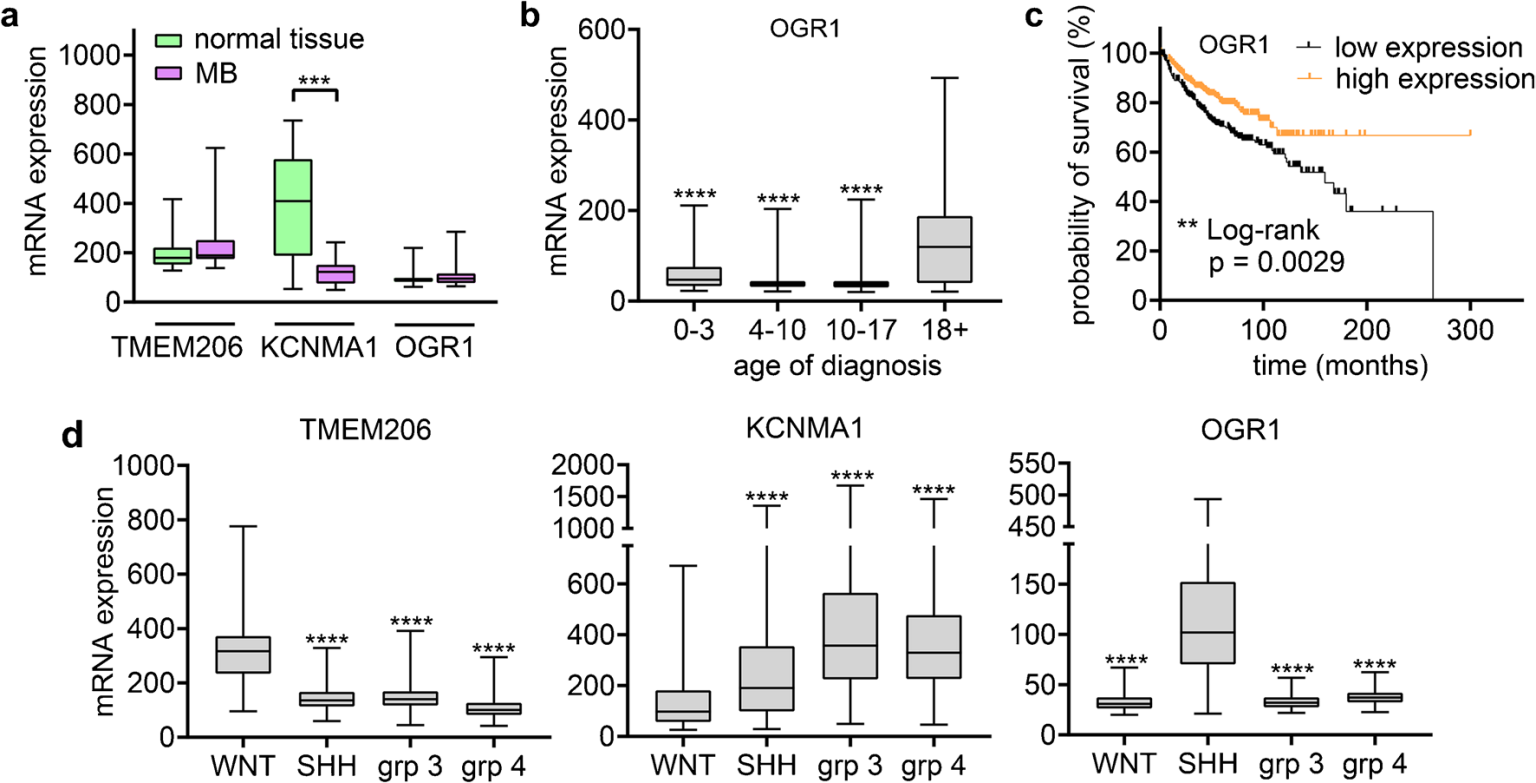
Expression of TMEM206, *KCNMA1, *and OGR1 in MB and Kaplan–Meier survival analysis***. ***(**a**) The Gump microarray dataset was used to determine the relative mRNA expression of TMEM206, *KCNMA1*, and OGR1 in normal tissue (*n* = 16) and MB (*n* = 19). (**b**) The Cavalli microarray dataset was used to determine the relative mRNA expression of OGR1 in patients with different ages at diagnosis (0–3: *n* = 132; 4–10: *n* = 349; 10–17: *n* = 181; 18 + : *n* = 101). (**c**) Kaplan–Meier survival curves of MB samples from the Cavalli microarray dataset with low (*n* = 318) or high (*n* = 314) mRNA expression of OGR1. The cutoff was determined using maximally selected ranked statistics in R (corresponding to the median). (**d**) The Cavalli microarray dataset was used to determine the relative mRNA expression of TMEM206, *KCNMA1* and OGR1 in the 4 molecular subgroups of MB (WNT: *n* = 70; SHH: *n* = 223; group 3: *n* = 144; group 4: *n* = 326). The microarray datasets were accessed on the 09.04.2023 through the GlioVis data portal for visualization and analysis of brain tumor expression datasets. Results are shown as box plots. Statistical analysis was performed with Mann–Whitney tests (**a**), Kruskal–Wallis tests (**b**, **d**), or a Log-rank (Mantel-Cox) test (**c**). *, *P* < 0.05; ***, *P* < 0.001; ****, *P* < 0.0001

Finally, we examined the expression of our genes of interest (GOI) in MB tissue from pediatric patients, using the Gump and Cavalli microarray datasets, which are freely available through the GlioVis database portal [7]. We compared the expression levels of the GOIs between MB and normal tissue using the Gump microarray dataset (Fig. 8a). While TMEM206 and OGR1 showed no significant alterations in their expression, *KCNMA1* mRNA levels were significantly lower in MB tissue (*P *< 0.0001). Previously, we reported that the expression of ASIC1a was higher in MB than in normal tissue [42]. When we sorted the data of the Cavalli microarray dataset by age of diagnosis, we observed that patients diagnosed as adults (18 + years of age) expressed significantly more OGR1 (*P* < 0.0001) (Fig. 8b). Next, we performed a survival analysis for the GOIs. Patients expressing high levels of OGR1 mRNA survived significantly longer than those expressing low levels (*P* = 0.0029) (Fig. 8c). In contrast, expression levels of ASIC1a, TMEM206, and KCNMA1 were not associated with changes in survival.

In the Cavalli microarray dataset, TMEM206 was particularly highly expressed in WNT tumors compared to other subgroups (*P* < 0.0001) (Fig. 8d). In contrast, *KCNMA1* had the highest expression in group 3 tumors and lowest expression in WNT. OGR1 was more highly expressed in SHH tumors than in the other 3 subgroups (*P* < 0.0001). ASIC1a expression is highest in type 4 and lowest in SHH MB [42]. Thus, there is no consistent expression pattern of the GOIs in different subtypes of MB.

## Discussion

In this study, we functionally identified three sensors of acidic pH_e_ in DAOY MB cells: ASICs, TMEM206, and OGR1. In contrast to the previously reported presence of ASIC1a and OGR1 [21, 42], the expression of functional TMEM206 in DAOY cells had not been documented until now. In addition, we found a prominent expression of BK_Ca_ channels, which were secondarily activated by acid via OGR1. The outwardly rectifying currents of TMEM206 and BK_Ca_ channels were most easily discerned at unphysiologically positive membrane potentials where strong acidification elicited currents with a complex pattern (Figs. 1 and 3). Nevertheless, also at the resting membrane potential, pH 5.0 elicited cell volume changes, which were sensitive to inhibitors of the three acid-sensors and of BK_Ca_ (Fig. 6), suggesting their contribution to the response of DAOY cells to strong acidification. Moreover, pH 4.5 even induced cell death, which was sensitive to the same inhibitors (Fig. 7).

Previous studies have already linked the activation of TMEM206 during strong acidic “shock” treatments to rapid changes in cell volume and cell death in HEK cells, HeLa cells, and neurons [39, 48, 51]. Like previously proposed for HEK cells [48, 51], we propose that at acidic pH, activation of ASICs and influx of Na^+^ ions lead to the depolarization of DAOY cells, increasing the open probability of TMEM206 and driving the influx of Cl^−^ ions. The coupled influx of Na^+^ and Cl^−^ ions would then increase the osmotic pressure and lead to cell swelling. While the activation threshold of TMEM206 is low at room temperature (~ pH 5.5), it increases at body temperature (~ pH < 6) [44], suggesting that cell death induction by activation of ASIC1a and TMEM206 might be relevant for a number of pathologic conditions associated with acidosis such as ischemic stroke or tumors. In addition, TMEM206 may be involved in other functions in MB cells, such as macropinosome shrinkage, an important mechanism for tumor growth [59]. Similarly, ASICs has other functions in tumor cells as well [16], for example it had been shown that in glioblastoma tumorspheres it activates a non-canonical necroptosis pathway [10]. DAOY cells evade this cell death by a low expression of RIP3, an important protein involved in the necroptosis pathway [42]. Our study suggests a possible novel function of ASIC1a in tumors, where it could synergize with TMEM206 to affect the cell volume. Suprisingly, OGR1-mediated activation of BK_Ca_ apparently also contributed to the initial swelling, albeit to a small degree (Fig. 6c). This was surprising, considering that K^+^-efflux is expected to lead to cell shrinking [24, 36].

To date, BK_Ca_ channels have not been studied in MB, but they have been extensively studied in other brain tumors, particularly in gliomas, where they may promote proliferation and migration [13]. Interestingly, the facilitation of migration through Ca^2+^-activated K^+^ channels is achieved largely by cell shrinking [8]. Furthermore, Ca^2+^-activated K^+^ channels such as BK_Ca_ and IK_Ca_ appear to be important for the volume regulation of glioma cells [35]. In DAOY cells, inhibition of BK_Ca_ reduced the slope of the secondary volume decrease at acidic pH (Fig. 6d), which was expected as BK_Ca_ channels allow K^+^ efflux. The delay by ~ 40 s of the volume decrease could at least in part be due to the fact that BK_Ca_ channels were activated with a similar delay (Figs. 1d and 3b), due to the delayed elevation of [Ca^2+^]_i_ by OGR1 activation (Fig. 5). Desensitization of ASIC1a within a few s in concert with activation of a K^+^ conductance would hyperpolarize the cells, now driving the efflux of Cl^−^ ions. Thus, we propose that Cl^−^-flux via TMEM206 contributes to the initial swelling as well as to the delayed shrinking of DAOY cells under strong acidification by “following” a leading cation, either Na^+^ (ASIC1a) or K^+^ (BK_Ca_). A role for TMEM206 in cell shrinking has previously also been found in HEK cells [48].

The decreased slope of the secondary shrinking of DAOY cells by inhibiting ASIC1a suggests that the initial swelling promoted subsequent shrinking, a process known as regulatory volume decrease (RVD). For example, swelling could activate TRP channels that increase Ca^2+^ [19]. Thus, in DAOY cells, ASIC1a and TMEM206 could be indirectly and TMEM206, OGR1, and BK_Ca_ directly involved in RVD. The partial reduction of cell shrinkage by blockade of either BK_Ca_ or TMEM206 suggests that activation of other Ca^2+^-activated K^+^ and Cl^−^ channels contributed to RVD as well. Thus, the changes in DAOY cell volume after acidic stimulation likely resulted from the complex interplay of several different ion channels and transporters. Irrespective of the exact mechanisms involved, the sustained decrease in cell volume shows that strong acidification leads to a dysregulation of volume homeostasis in DAOY cells.

Two hours exposure to a strongly acidic environment resulted in the death of the majority of DAOY cells (Fig. 7), which was reduced by the inhibition of ASIC1a, TMEM206, BK_Ca_, or OGR1. On the one hand, the short time frame suggests a necrosis-like cell death mechanism. A link between cell swelling and necrosis, a process known as necrotic volume increase (NVI), has been repeatedly documented in other studies [5, 37], and TMEM206 has been previously associated with cell volume alterations and cell death in HeLa cells, HEK cells, and neurons [39, 48, 51]. On the other hand, the delayed shrinking of DAOY cells at pH 4.5 argues against NVI and is rather reminiscent of apoptotic volume decrease. Inhibition of BK_Ca_ by IbTx indeed almost completely reversed increased cell death at acidic pH, suggesting that volume decrease played a major role. The incomplete reversal of cell death by OGR1 inhibition (Fig. 7b) further suggests that other mechanisms contributed to Ca^2+^ increase and activation of BK_Ca_. The fact that IbTx had a stronger effect on cell death than on volume changes suggests that the role of BK_Ca_ in DAOY cell death was not limited to mediating cell shrinking. Thus, whether and to which extent the cell volume changes were related to cell death and whether both phenomena occur in MB in situ is unclear and requires further investigation.

While DAOY cells serve as a model for SHH MB, UW228 are a model for WNT MB [18]. SHH MB is characterized by an increased expression of *ASIC1a* and a reduced expression of ASIC2 and this expression pattern is recapitulated in DAOY cells, suggesting their suitability for studying the role of ASICs in SHH MB [42]. In contrast, ASICs are absent in UW228 cells, rendering them an unsuitable model for studying the role of ASICs in MB [42] (Table 1). In the current study, we found that DAOY and UW228 cells both expressed TMEM206, but the mRNA expression and current amplitude of TMEM206 was ~ twofold lower in UW228 than in DAOY cells. Overall, TMEM206 exhibits similar expression level in MB and normal tissue (Fig. 8a), but its expression is more pronounced in WNT than SHH MB (Fig. 8d). Thus, the lower expression of TMEM206 in UW228 than in DAOY cells does not recapitulate the distinct expression patterns observed in the two MB subtypes (Table 1).

**Table 1:** qualitatively summarizes the expression of the GOIs in SHH MB and WNT MB, and in UW228 and DAOY cells. Expression data for MB tissue is based on the Cavalli dataset, expression data for the cells on functional data and qPCR. Data for ASIC1a are from [42]. “ + ” denotes normal expression, “- “ downregulation” and “ +  + ” upregulation

	ASIC1a	TMEM206	BK_Ca_	OGR1
WNT MB	+ +	+ +	+	+
UW228	-	+	+	-
SHH MB	+ +	+	+ +	+ +
DAOY	+ +	+ +	+ +	+ +

A possible involvement of BK_Ca_ in MB tumor biology is supported by the fact that BK_Ca_ is considerably less expressed in MB compared to normal tissue (Fig. 8a). Like TMEM206, both DAOY and UW228 cells expressed BK_Ca_, but the mRNA expression and current amplitude were ~ tenfold lower in UW228 cells than DAOY cells. Interestingly, WNT tumors exhibited the lowest amount of *KCNMA1* mRNA compared to more aggressive MB subgroups (Fig. 8d). Thus, the lower expression of BK_Ca_ in UW228 than in DAOY cells accurately reflects the distinct expression patterns in the two MB subtypes (Table 1).

The analysis of the Cavalli microarray dataset revealed that OGR1 is highly expressed in SHH MBs compared to other MB subgroups (Fig. 8d). Our finding that DAOY cells strongly express OGR1, while UW228 cells do not (Fig. 5), suggests that DAOY cells are representative for SHH MB concerning OGR1 expression (Table 1). In a stem cell-rich DAOY MS culture, the expression of OGR1 increased even further (threefold). The role of OGR1 in cancer stem cells remains unknown. Surprisingly, the survival rate of MB patients expressing high levels of OGR1 was increased in the Cavalli microarray dataset (Fig. 8c). Previously, it was shown that OGR1 activates the MEK/ERK pathway in DAOY cells through acid-induced elevation of [Ca^2+^_i_] [21], which could promote proliferation and tumor growth. Additionally, OGR1 increases expression of TRPC4 channels in DAOY cells [54]. In the same study, direct activation of TRPC4 channels promoted migration, however indirect activation through acid-induced OGR1-activation did not. Moreover, when investigating the effects of OGR1 on other cancer types, conflicting data emerge depending on the specific tumor type. While some studies suggest that OGR1 exhibits pro-tumor effects, for instance in melanoma, pancreatic cancer and colorectal cancer [20, 32, 56], others suggest the opposite [31, 45, 60]. Given that MB patients expressing high levels of OGR1 exhibit a higher survival rate (Fig. 8c), the role of OGR1 in MB might be anti-tumoral. However, the high expression could also be an epiphenomenon that is not causally linked to the survival. Nevertheless, the high expression of OGR1 in adult MB patients (Fig. 8b) was striking, because MB in adult patients is very rare and about 75% of adult MBs belong to the SHH group and metastasis almost never occurs [29]. Therefore, the potential anti-tumoral role of OGR1 in SHH MB merits further investigation. The high expression of both BK_Ca_ and OGR1 in DAOY cells (Table 1) indicates that DAOY cells are a suitable model for studying their functional relationship in SHH MB.

In summary, we characterized the acid sensors ASIC1a, TMEM206, OGR1 and BK_Ca_ channels in MB cells and our results indicate that DAOY cells are a valuable model to study their functional interrelationship, including their role in volume regulation and cell death.

## Materials and methods

### Materials

PcTx1 and IbTx (Smartox Biotechnology, Sainte-Égrève, France), YM-254890 (Biozol, Eching, Germany), DIDS (Sigma-Aldrich, St. Louis, MI, USA), bepridil hydrochloride (MedChemExpress, Monmouth Junction, New Jersey, United States), NS-11021 (Alomone Labs, Jerusalem, Israel), and pregnenolone sulfate (Sigma-Aldrich) were purchased in research quality.

### Cell culture of adherent DAOY and UW228 cells

DAOY cells [23] and UW228 cells [27] were kindly provided by G. Ciarimboli (Münster, Germany); UW228 cells were originally provided by John R. Silber (University of Washington, Seattle, USA). DAOY cells were cultured in Eagle’s minimal essential medium (MEM Eagle; PAN-Biotech, Aidenbach, Germany) supplemented with 10% fetal bovine serum (FBS; Sigma-Aldrich). UW228 cells were cultured in Dulbecco’s modified Eagle medium (DMEM)/F12 supplemented with 10% FBS and 2 mM L-Glutamine (Thermo Fisher Scientific). Cells were maintained at 37 °C with 5% CO_2_ and passaged every 3–4 days with 0.25% trypsin–EDTA solution (Thermo Fisher Scientific).

### Cell culture of medullospheres

DAOY cells were cultured as medullospheres in suspension according to the protocol by Gong et al. [15]. First, adherent DAOY cells were allowed to become confluent in a 10 cm petri dish (Sarstedt AG & Co. KG, Nümbrecht, Germany). Next, they were detached with 0.25% trypsin–EDTA, washed and centrifuged two times with DPBS (PAN-Biotech) and resuspended in medullosphere culturing medium, which contained DMEM/F-12 and the following supplements: 1% N-2 supplement (Gibco, Thermo Fisher Scientific), 2% B-27 supplement (PAN-Biotech), 20 ng/ml recombinant human fibroblast growth factor (FGF; 154 a.a.; Thermo Fisher Scientific) and 20 ng/ml recombinant human epidermal growth factor (EGF; R&D Systems, Minneapolis, USA). The cells were resuspended and transferred into a T75 Nunc™ non-treated flask (Thermo Fisher Scientific). Medullosphere culturing medium was added to the flask to a total of 20 ml. Cells were cultured for 7 d and then harvested for use in RT-qPCR.

### Whole-cell patch clamp

DAOY cells were seeded on glass coverslips and allowed to attach for at least 1 h in the incubator. Next, the coverslips were mounted in a perfused bath on the stage of an inverted microscope (IX71, Olympus) and kept at RT.

The conditioning bath solution contained (in mM): NaCl 115, KH_2_PO_4_ 0.4, K_2_HPO_4_ 1.6, D-glucose 5, MgCl_2_ 1, Na^+^ gluconate 25, Ca^2+^ gluconate 3, HEPES 5. pH was adjusted to 7.3 with NaOH and HCl. The low Cl^−^ bath solution (Fig. 2b, c) contained: NaCl 5, KH_2_PO_4_ 0.4, K_2_HPO_4_ 1.6, D-glucose 5, MgSO_4_ 1, Na^+^ gluconate 140, Ca^2+^ gluconate 8, HEPES 5. pH was adjusted to 7.3 with NaOH and H_2_SO_4_. For acidic bath solutions, HEPES was replaced by MES; pH was adjusted with NaOH and HCl (control) or H_2_SO_4_ (low Cl^−^).

Patch-clamp experiments were performed in the whole-cell configuration. Patch pipettes had an input resistance of 5–7 MΩ, when filled with an intracellular-like solution containing (in mM): K^+^ gluconate 95, KCl 30, NaH_2_PO_4_ 1.2, Na_2_HPO_4_ 4.8, D-glucose 5, MgCl_2_ 2.38, EGTA 1, Ca^2+^ gluconate 0.726; pH was adjusted to 7.2 with KOH and HCl. The intracellular-like solution used to suppress outwardly rectifying K^+^ currents (Figs. 1a-c, 4a) contained (in mM): D-gluconic acid (40–50% w/w) 107.38, CsOH 107.38, CsCl_2_ 12.62, sodium gluconate 6, D-glucose 5, MgCl_2_ 2.38, EGTA 1, Ca^2+^ gluconate 0.726, HEPES 10. pH was adjusted to 7.2 with CsOH and D-gluconic acid (40–50% w/w).

Currents were recorded using a patch-clamp amplifier (Axopatch 200 B), the Axon-CNS (Digidata 1440 A) and Clampex software (Molecular Devices). Data were filtered at 1 kHz with a low-pass filter and was analyzed with the PCLAMP software. The sampling rate was 20 kHz. No correction of liquid junction potentials was performed.

### Ratiometric Ca^2+^*-*imaging

To determine intracellular Ca^2+^-concentrations, DAOY cells were seeded on cover slips, mounted in a cell chamber, and perfused with conditioning and acidic bath solutions as described for whole-cell patch clamp.

Fluorescence was measured every 2 s on an inverted microscope (IX71, Olympus, Chromaphor) using a Fluar 20 × /0.75 objective (Olympus) and Till Vision real-time imaging software (Till Photonics). Cells were loaded for 30 min at 37 °C with 2 μM Fura-2-AM (Molecular Probes) in the bath solution. Fura-2 was excited at 340/380 nm and the emission was recorded between 470 and 550 nm using a sensicam CCD camera (PCO imaging). Acquisition and data analysis were done using the Till Vision software and Excel. Ionomycin (Santa Cruz Biotechnology, Dallas, USA) was used as a positive control.

### Reverse transcription quantitative PCR

Adherent DAOY and UW228 cells were cultured for 48 h prior to RNA isolation at pH 7.4. DAOY cells in suspension were cultured for 7 days in an MS culture prior to RNA isolation. Total RNA was isolated using NucleoSpin RNA isolation kit (Macherey–Nagel, Düren, Germany). Concentration and quality of the RNA was measured using a NanoDrop 2000c spectrophotometer (Thermo Fisher Scientific). RNAs with a 260 nm/280 nm ratio > 2.00 and a 260 nm/230 nm ratio > 1.80 were used for reverse transcription. RNA was reverse transcribed to cDNA using the High-Capacity cDNA Reverse Transcription Kit (Thermo Fisher Scientific).

For reverse transcription quantitative real-time PCR (RT-qPCR), each reaction contained 1 µl cDNA (20 ng cDNA in 1 μl H_2_O), 1 µl AM-MGB labelled hydrolysis probe (TaqMan™; Thermo Fisher Scientific), 5 µl Luna Universal qPCR Master Mix (New England Biolabs, Ipswich, USA) and 3 μl H_2_O. For human DAOY and UW288 cells, the following TaqMan™ probes were used: HPRT1 (Hs02800695), GPR68 (Hs00268858_s1), *KCNMA1* (Hs00266938_m1), TMEM206 (Hs01558462_m1). A sample without cDNA served as negative control. Each reaction was pipetted into 4-Strip 0.1 ml Tubes (STARLAB, Hamburg, Germany) and transferred to the Rotor-Gene Q thermocycler (Qiagen, Hilden, Germany) for measurement. Reactions were performed in technical triplicates for each biological replicate, with technical duplicate negative controls for each TaqMan™ probe. qPCR was started with a denaturation phase (180 s, 95°), followed by 40 cycles of denaturation (30 s, 95 °C), annealing (20 s, 60 °C) and extension (20 s, 72 °C). Experiments were repeated with RNA isolated from *n* = 3 independent cell batches and analyzed using the ΔΔCt method. Efficiency of the house keeping genes HPRT1 [11] was determined by a standard curve and was close to 100%.

### Cell volume measurements

The relative changes in the cell volume of DAOY cells were measured (Fig. 6) with a modified protocol by Altamirano et al. [2]. First, the cells were loaded with 2 μM Fura-2 and seeded on cover slips as described in the section “Ratiometric Ca^2+^-imaging”. They were perfused with either a conditioning (pH 7.4) or an acidic solution (pH 5.0) for 10 min. The solutions were made as described in the section “Whole-cell patch clamp”. Fluorescence was measured every 10 s on an inverted microscope (IX71, Olympus, Chromaphor) using a Fluar 20 × /0.75 objective (Olympus) and Till Vision real-time imaging software (Till Photonics). Fura-2 was excited at its isosbestic wavelength (358 nm), and the emission was recorded between 470 and 550 nm using a sensicam CCD camera (PCO imaging). At the isosbestic wavelength, Fura-2 is insensitive to Ca^2+^ and the cell volume measurements are not altered by changes in [Ca^2+^]_i_. When a cell swells, the intracellular Fura-2 is diluted and its fluorescent signal is reduced, and vice versa when the cell shrinks. Acquisition and data analysis were done using the Till Vision software and Excel. The hypotonic solution used to confirm the validity of this method (Fig. 6a) contained (in mM): NaCl 95, KH_2_PO_4_ 0.4, K_2_HPO_4_ 1.6, D-glucose 5, MgCl_2_ 1, Ca^2+^ gluconate 1.3. pH was adjusted to 7.4 with NaOH and HCl.

### Propidium iodide staining

2 × 10^4^ DAOY cells per experimental condition were collected in a 1.5 ml Eppendorf tube and washed once with 1 ml DPBS. The cells were spun down for 1.5 min at 1 200 rpm. DPBS was removed and the cells were resuspended in 600 μl of the acid shock solution. This solution contained (in mM): NaCl 145, KCl 5, MgCl_2_ 1, CaCl_2_ 2, D-glucose 10. pH was adjusted to pH 7.4 or 4.5 with NaOH and HCl. 10 mM HEPES was added for pH 7.4 solutions and 10 mM MES was added for pH 4.5 solutions. The tubes were placed with an open lid in an incubator at 37 °C with 5% CO_2_ for 1 h and 45 min. Afterwards, the cells were gently mixed with a pipette and distributed in a 96-well plate (100 μl per well). Each condition was measured with 5 replicates, corresponding to 5 wells, and repeated in 3 independent experiments. 1 μg of PI (Thermo Fisher Scientific) was added to each well and cells were incubated for another 10 min. Using an inverted microscope (IX71, Olympus, Chromaphor) with a sensicam CCD camera (PCO imaging), transmission images from the cells were acquired. The cells were excited with green light (555 nM) and fluorescent images were acquired. The total number of cells was determined by counting the cells in the transmission images using ImageJ. The number of PI-positive cells was determined by counting the cells in the fluorescent images. The relative number of PI-positive cells in % was then calculated by dividing the number of cells counted in the fluorescent images with the ones counted in the transmission images.

### Statistical analysis

Data are reported as mean ± SD or as box plots with whiskers, showing the minimal and maximal values, respectively. Statistical analyses were performed in Microsoft Excel 2007 and Prism 10.1.2, with significance threshold set to *p* ≤ 0.05. For the analysis of RT-qPCR and whole-cell patch clamp experiments, we used parametric tests - paired Student’s t-tests or one-way/repeated-measures ANOVAs. For the analysis of Ca^2+^-imaging, cell volume measurements, and PI-stainings, we used nonparametric tests - Wilcoxon signed-ranked tests and Kruskal–Wallis tests. GlioVis microarray datasets were analyzed with Mann–Whitney tests, Kruskal–Wallis tests, and a Log-rank (Mantel-Cox) test.

## Data Availability

Datasets generated and analysed during this study are included in this published article. Additional information is available from the corresponding author upon reasonable request.
